# New insights into markers for distinguishing neuroendocrine prostate cancer: evidence from single-cell analysis

**DOI:** 10.3389/fimmu.2025.1551815

**Published:** 2025-03-14

**Authors:** Hailang Luo, Boyang Li, Meng Zhang, Hongqun Wang, Zongyao Hao, Qintao Ge, Chaozhao Liang

**Affiliations:** ^1^ Department of Urology, the First Affiliated Hospital of Anhui Medical University, Anhui Medical University, Hefei, Anhui, China; ^2^ Institute of Urology, Anhui Medical University, Hefei, Anhui, China; ^3^ Anhui Province Key Laboratory of Urological and Andrological Diseases Research and Medical Transformation, Anhui Medical University, Hefei, Anhui, China; ^4^ Department of Pathology, the Third People's Hospital of Bengbu City, Bengbu, China; ^5^ Department of Urology, Fudan University Shanghai Cancer Center, Shanghai, China; ^6^ Department of Oncology, Shanghai Medical College, Fudan University, Shanghai, China

**Keywords:** neuroendocrine prostate cancer (NEPC), single cell analysis, biomarker, WDFY4, ASCL1

## Abstract

**Background:**

Neuroendocrine prostate cancer (NEPC) is a highly aggressive malignancy with few effective treatment options. The identification of reliable biomarkers for NEPC is essential for early detection and intervention.

**Methods:**

We combined single-cell and bulk transcriptome analysis to identify novel markers of NEPC. InferCNV to assess copy number variations and leveraging consensus non-negative matrix factorization (cNMF) to characterize transcriptional programs. Pseudotime analysis was used to decipher prostate cancer (PCa) progression differentiation trajectory. BayesPrism integrates single-cell results and TCGA-PRAD sequencing information to further study prognostic features. Immunohistochemistry (IHC) was performed to validate the elevated expression of ASCL1 and WDFY4 in NEPC.

**Results:**

We identified five distinct expression programs of PCa malignant epithelial cells, where Module 3 presented NEPC expression patterns, with activation of DNA replication and cell cycle pathways and classical NEPC marker expression. Patients with high Module 3 proportion correlated to poor clinical outcomes, advanced Gleason scores, and higher T stages. Pseudotime analysis highlighted key trajectory-dependent genes involved in the transition to NEPC, where expression of ASCL1 and WDFY4 elevated with progressing to NEPC cell fate, which were further confirmed by IHC analysis, indicating that WDFY4 and ASCL1 might be novel potential markers for distinguishing NEPC.

**Conclusions:**

Combined single-cell and bulk analysis, we highlight the cellular heterogeneity and transcriptional programs, validated novel biomarkers of NEPC. Providing a foundation for early prediction of NEPC and management.

## Introduction

Castration-resistant prostate cancer (CRPC) poses a significant challenge in the management of advanced prostate cancer (PCa), as it is characterized by tumor progression despite androgen deprivation therapy (ADT) (1). Approximately 10-20% of patients with PCa progress to CRPC, with median survival rates for these patients ranging from 9 to 30 months (2). This stage of PCa is often associated with limited therapeutic options and poor clinical outcomes (3). NCCN guidelines for CRPC recommend treatments including abiraterone, enzalutamide, apalutamide, darolutamide, or docetaxel (4). Although the range of available treatment options is gradually expanding and patient survival is improving, CRPC remains a highly lethal malignancy overall. Despite significant advances in understanding the molecular mechanisms underlying PCa, the factors driving CRPC progression and the associated drug resistance are still not fully elucidated. A more comprehensive understanding of the molecular basis and cellular heterogeneity of CRPC is essential for identifying novel therapeutic targets and enhancing patient prognosis (5).

The prevalence of neuroendocrine prostate cancer (NEPC) is anticipated to rise as patients undergo multiple therapies (1). Neuroendocrine tumor cells can be histologically distinguished from other cell types within the complex PCa microenvironment or using the circulating tumor DNA (ctDNA) (6); however, their scarcity, particularly in the early stages of the disease, often leads to the misdiagnosis of early-stage NEPC (7). Currently, the diagnosis of NEPC primarily relies on the immunohistochemical analysis of several biomarkers—specifically, androgen receptor (AR) negativity, elevated MKI67 expression, and positivity for neuroendocrine markers such as chromogranin A (CHGA), synaptophysin (SYP), enolase 2 (ENO2), and neural cell adhesion molecule 1 (NCAM1). Nevertheless, the heterogeneous expression of these proteins in NEPC tumor cells significantly compromises their diagnostic sensitivity (8, 9).

The emergence of next-generation sequencing technologies has facilitated a comprehensive exploration of the molecular characteristics associated with NEPC. Key factors contributing to NEPC have been identified, particularly mutations in FOXA1 and SPOP found in primary prostate cancer (PCa), lineage plasticity resulting from RB1 loss and TP53 dysfunction, as well as the activation of the polycomb repressive complex 2 (PRC2), including components such as EZH2, in advanced PCa cases (10). Furthermore, these investigations have revealed over ten gene sets associated with NEPC, collectively encompassing thousands of differentially expressed genes (DEGs). However, these genomic assessments exhibit significant variability, which can be attributed to several factors: a) the research is primarily based on a small cohort of NEPC cases (11); b) the gene expression profiles of CRPC-adenocarcinoma closely resemble those of NEPC (7); and c) the gene sets largely rely on transcriptomic data obtained from a diverse array of tumors rather than being exclusively derived from NEPC tumor cells (12). Consequently, there is an urgent need to establish sensitive and specific biomarkers for NEPC to enhance both fundamental research and clinical applications (13).

In this study, we analyzed the distinct transcriptional patterns observed in primary and castration-resistant prostate cancer, illustrating their developmental trajectories. During the progression from primary prostate cancer to castration-resistant prostate cancer, the expression levels of certain genes progressively increased, suggesting their potential as biomarkers for castration-resistant prostate cancer and warranting further investigation.

## Materials and methods

### Data acquisition

The single-cell transcriptomic data were sourced from the PRJNA699369 database, associated with the published study (14). This study identified a small population of cells within primary prostate cancer that exhibit CRPC characteristics even prior to hormonal therapy. These cells, inherently possess castration resistance rather than developing it as an adaptive response to hormonal therapy, are linked to biochemical recurrence and distant metastasis. Data integration across samples was performed using the R package Seurat (version 4.3.0), which was utilized to construct Seurat objects and annotate grouping information for each sample (15). Clinical profiles and corresponding gene expression data were obtained from The Cancer Genome Atlas (TCGA, https://portal.gdc.cancer.gov/), employing the R package TCGAbiolinks (version 2.22.0) to retrieve TCGA-PRAD data (16). Gene expression levels across all three groups were expressed in transcripts per million (TPM). Messenger RNAs with a TPM value below 1 in over 90% of samples were considered background noise and were excluded from further analysis. The primary endpoint of this study was progression-free survival (PFS) (17).

### Processing of single-cell transcriptomics data

Raw data preprocessing was performed using the Seurat package, which involved excluding cells with fewer than 200 or more than 2500 detected transcripts, as well as those with mitochondrial gene percentages exceeding 10%. To address cell cycle effects on single-cell transcriptomic data, the CellCycleScoring function in Seurat was used for cell cycle scoring. The NormalizeData function, using the LogNormalize method, was applied for logarithmic transformation and normalization. Highly variable genes were identified using the FindVariableFeatures function with the variance stabilizing transformation (vst) method, retaining the top 2000 genes with the highest variability. Batch effects between samples were subsequently removed using the ScaleData function. Following data preprocessing, dimensionality reduction was performed to facilitate further analysis of high-dimensional single-cell transcriptomic data. Principal component analysis (PCA), the most widely adopted method for this purpose, was applied using the RunPCA function in Seurat, retaining the top 50 principal components. Next, the FindNeighbors function was employed to identify K-nearest neighbor (KNN) relationships among cells, and cell clustering was implemented using the Louvain method via the FindClusters function. Annotation of cell clusters was manually conducted based on established signature markers (14, 18, 19).

### Identification of tumor cells by InferCNV analysis

The formation of polyploid or aneuploid cells is a pathological hallmark of malignancy, characterized by copy number variations (CNV). The inferCNV (https://github.com/broadinstitute/inferCNV) was utilized to analyze copy number variations (CNVs) in scRNA-seq data. InferCNV facilitates the visualization of CNVs in cells based on single-cell RNA-Seq expression data. The initial CNV estimates are derived by analyzing the genes, including their chromosomal locations, and averaging their relative expression values (20, 21). Cell types were initially classified using the Seurat package, after which InferCNV was applied to calculate CNVs across all autosomes for each cell type. For the 10× Genomics single-cell data, a cutoff value of 0.1 was utilized.

### Uncovering diverse gene expression patterns among malignant cells in PCa

To uncover transcriptional programs in malignant epithelial cells, we utilized consensus Non-Negative Matrix Factorization (cNMF) through the cNMF module from the omicverse package (version 1.6.4) (22). This unsupervised methodology decomposed gene expression data into metagenes that represent various transcriptional states. Prior to the analysis, we preprocessed the gene expression data to concentrate on genes exhibiting high variability among malignant cells. Subsequently, we performed cNMF, determining the optimal number of factors (k) through an iterative method that maximized the cophenetic correlation coefficient, thereby ensuring a robust and biologically relevant factorization. The resulting metagenes signify transcriptional states characteristic of malignant epithelial cells, facilitating further investigation into their potential functional implications in tumor biology.

### Trajectory inference analysis

To investigate cellular differentiation trajectories, we conducted pseudotime analysis using the Monocle2 package (version 2.22.0) (23). Monocle2 enables the reconstruction of lineage trajectories based on single-cell gene expression data. The gene expression matrix was initially preprocessed by filtering out low-quality cells and genes, followed by normalization and variance stabilization. Highly variable genes were selected to ensure robust trajectory inference. Monocle2 was then used to order cells along a pseudotime axis based on their transcriptional profiles, applying the DDRTree method for dimensionality reduction and trajectory construction. This approach facilitates the identification of branching points, which represent potential cellular decision-making events during differentiation.

### Enrichment analysis

To further uncover the different biological functions of these genes patterns, enrichment analysis was conducted, and annotated based on GO, KEGG, WiKipathways databases. In addition, we also conducted GSEA. All of the enrichment analysis performed on scRNA data were used SCP pipeline(https://github.com/zhanghao-njmu/SCP).

### BayesPrism deconvolution analysis

To deconvolute bulk RNA sequencing data into distinct cell types, BayesPrism algorithm was conducted. BayesPrism act as a framework driven by models that probabilistically aims to distinguish bulk gene expression data through the use of reference single-cell RNA sequencing datasets. This approach incorporates a Bayesian model that efficiently reduces the noise found in batch expression data, while also tackling the fundamental uncertainty associated with single-cell reference datasets (24).

### IHC analysis

Prostate tissues from patients (including 2 cases of primary PCa and 2 cases of NEPC) who underwent radical prostatectomy in the Department of Urology at the First Affiliated Hospital of Anhui Medical University were selected for this analysis. We conducted IHC staining to evaluate the expression of NSE (NSE Polyclonal antibody, Cat# 10149-1-AP, RRID: AB_2099180, Proteintech, USA), CHGA (Chromogranin A Polyclonal antibody, Cat# 23342-1-AP, RRID: AB_2879259, Proteintech, USA), ASCL1(ASCL1 Polyclonal antibody, Cat# 23751-1-AP, RRID: AB_2935459, Proteintech, USA) and WDFY4 (WDFY4 Polyclonal antibody, Cat# 17558-1-AP, RRID: AB_2288447, Proteintech, USA). Detailed IHC procedures could refer to our prior studies (20, 21). Tumor samples were gathered and preserved in a 4% formaldehyde solution for 24 hours. Subsequently, these samples were embedded in paraffin and sectioned into approximately 5 μm thick slices. The tumor sections underwent deparaffinization and rehydration, followed by the inhibition of endogenous peroxidase activity and antigen retrieval. After that, a 5% BSA solution was applied to the tumor sections to minimize non-specific binding for 30 minutes, after which they were incubated with primary antibodies overnight (dilution ratio 1:200). Following incubation with goat anti-rabbit IgG-HRP (1:200, GB23303, Servicebio, China) as the secondary antibody for one hour, the tumor sections were visualized using a DAB kit.

### Statistical analysis

Statistical analysis, data processing, and visualization were conducted using R software (version 4.2.2) and Python (version 3.9). Group differences were evaluated with Kruskal-Wallis and Wilcoxon tests, while the Chi-square test was employed to compare clinical characteristics across groups. Two-tailed p-values were calculated with significance set at *p* < 0.05.

## Results

### Cell population characterization of prostate cancer samples

**Figure 1:**
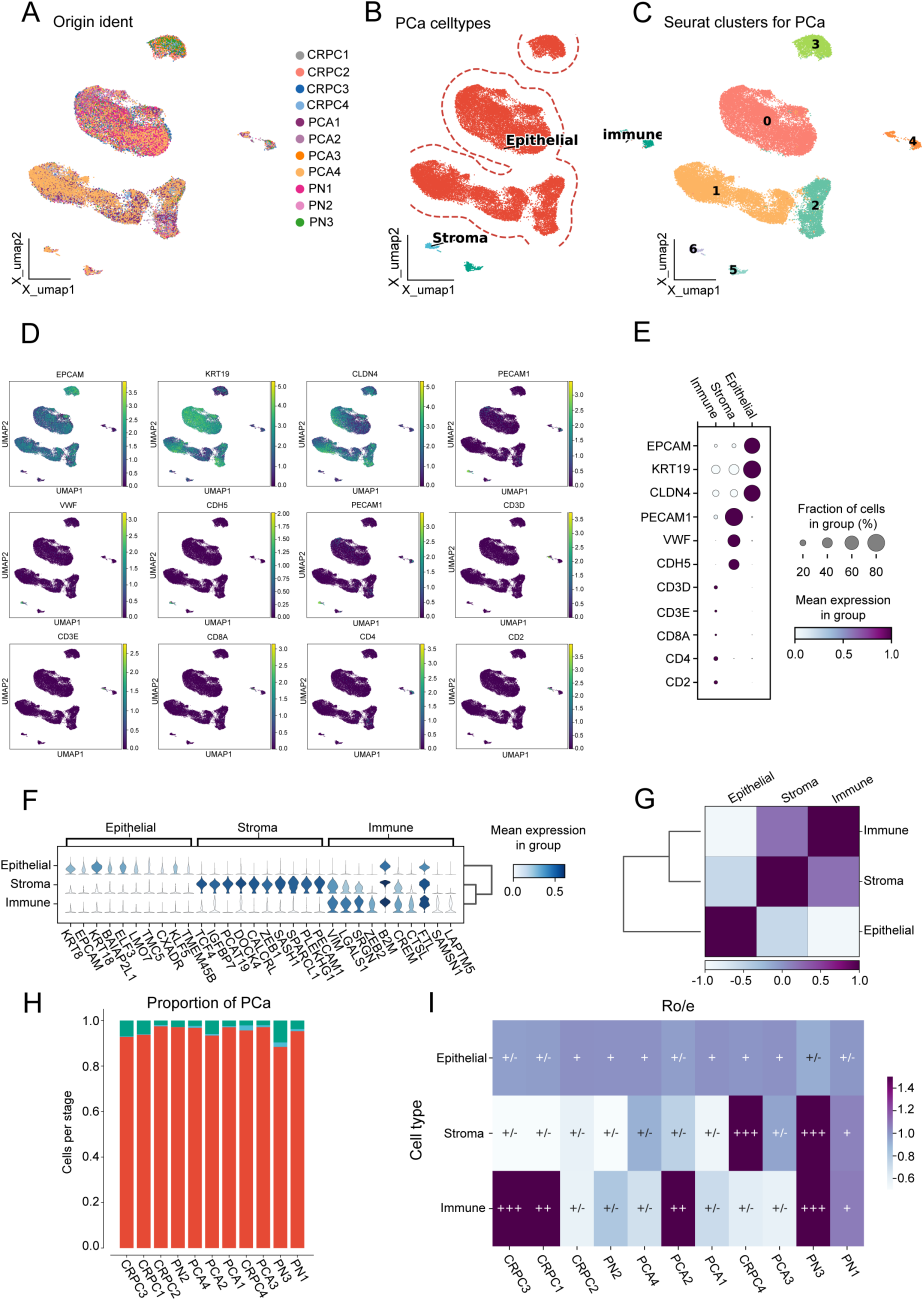
Cell population characterization in prostate cancer samples. **(A)** Uniform manifold approximation and projection (UMAP) visualization of 11 prostate cancer (PCA) samples. **(B)** UMAP visualization distinguishing three major cell types. **(C)** UMAP visualization showing seven distinct clusters. **(D)** UMAP visualization displaying marker gene expression patterns. **(E)** Dot plot illustrating marker gene expression across the three cell types. **(F)** Violin plots showing mean expression values of differentially expressed genes among the three cell types. **(G)** Heatmap depicting correlation between epithelial, stromal, and immune cell populations. **(H)** Proportion of each cell type across the 11 prostate cancer samples. **(I)** Ro/e scores indicating tissue preference distribution for the three cell types across the 11 samples.

After removing batch effects (**Figure 1A**), we identified seven clusters (**Figure 1B**), which were subsequently annotated into three primary cell populations (**Figure 1C**). The expression of marker genes for canonical epithelial, stromal, and immune markers was examined. Key markers, including EPCAM, KRT19, and CLDN4, were found to be enriched in epithelial cells; PECAM1 and VWF were enriched in stromal cells; and CD3, CD8A, and CD4 were prominent in immune cell populations. A dot plot illustrating marker gene expression across these cell types confirmed distinct expression patterns (**Figures 1D, E**). Furthermore, differentially expressed genes among the three clusters were identified (**Figure 1F**). Mean expression values across the three cell types indicated higher levels of KRT19 and EPCAM in epithelial cells, PECAM1 and VWF in stromal cells, and CD3D, CD8A, and CD4 in immune cells, thereby supporting robust annotation. Moderate positive correlations were observed between epithelial and stromal cells, while immune cells exhibited distinct expression patterns with lower correlations to either epithelial or stromal populations (**Figure 1G**). The proportion of each cell type across prostate cancer samples was quantified (**Figure 1H**), and the tissue preference of each cluster was assessed using Ro/e scores (**Figure 1I**).

### Five distinct expression patterns of malignant epithelial cells were identified

**Figure 2:**
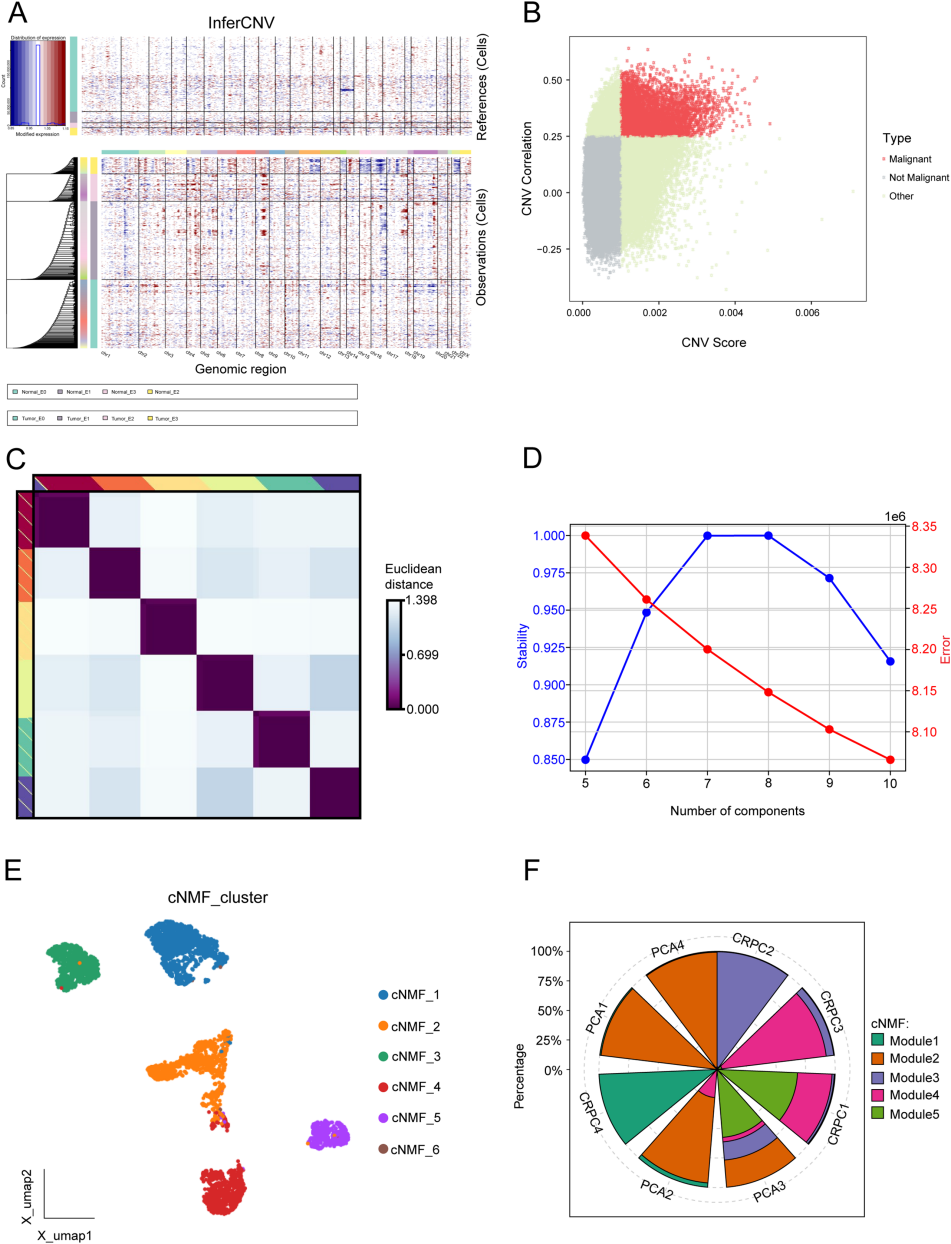
Comprehensive analysis of malignant programs. **(A)** Inferred large-scale copy number variations (CNVs) distinguishing cancer cells (blue) from non-cancer cells (red). Chromosomal regions are displayed on the x-axis, while tumor and normal cells are represented on the y-axis. **(B)** Scatter plot of CNV correlation versus CNV score, categorizing cells into malignant, non-malignant, and other groups. **(C)** Consensus heatmap illustrating Euclidean distances between transcriptional programs, highlighting distinct clusters of cells based on gene expression profiles. **(D)** Evaluation of cNMF model stability and error rates across different component numbers, selecting an optimal component number based on high stability and low error rate. **(E)** UMAP visualization of the six malignant transcriptional programs. **(F)** Proportion of five transcriptional modules across eight prostate cancer samples.

InferCNV was applied to assess CNVs across genomic regions in malignant and non-malignant cells. The heatmap (**Figure 2A**) illustrates CNV patterns, with red indicating amplifications and blue indicating deletions. The top panel corresponds to reference (non-malignant) cells, while the bottom panel represents observed malignant cells, revealing distinct chromosomal alterations across multiple genomic regions. A scatter plot of CNV correlation versus CNV score (**Figure 2B**) was used to classify cells into malignant, non-malignant, and other groups.

To identify transcriptional programs in malignant epithelial cells, we employed cNMF. A consensus heatmap (**Figure 2C**) visualizes the Euclidean distance between transcriptional programs, revealing distinct clusters of cells based on gene expression profiles. The stability and error rate of the cNMF model were evaluated across different component numbers (**Figure 2D**), with the optimal number of components selected based on high stability and low error rates. Six malignant programs were initially identified (**Figure 2E**). Due to the limited cell count in Module 6, it was excluded from further analyses, resulting in five key transcriptional programs. The proportions of these modules differed significantly across samples. Module 2 represents a cell population specific to primary prostate cancer, while Module 3 is CRPC. Modules 1, 4, and 5 are present in both groups, potentially representing pre-existing latent CRPC cells (**Figure 2F**).

### Functional enrichment and gene module characterization

**Figure 3:**
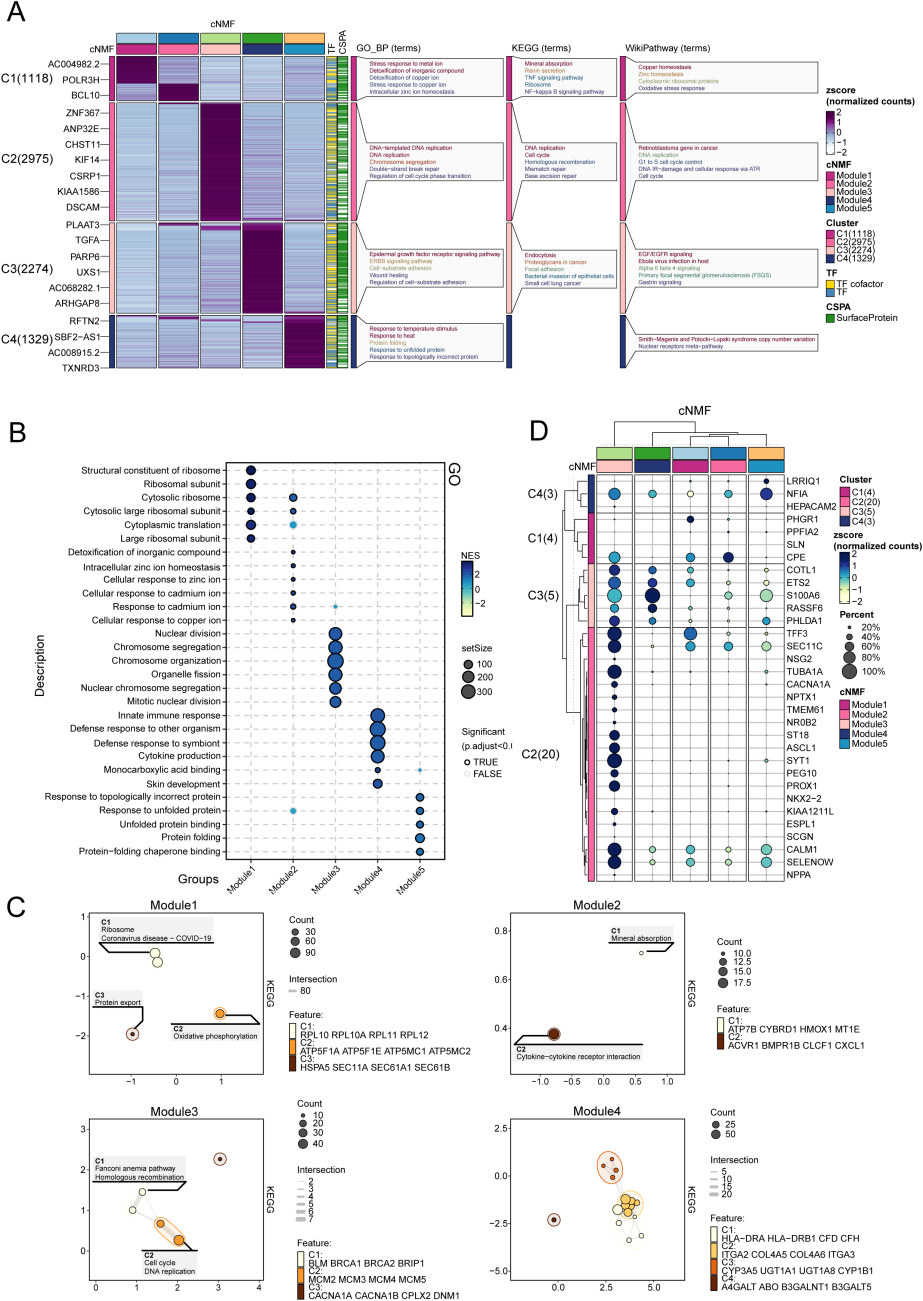
Functional enrichment and gene module characterization. **(A)** Expression patterns and associated biological functions and pathways of representative genes across the five transcriptional programs, based on GO, KEGG, and WikiPathways databases. **(B)** GO enrichment analysis for Modules 1–5, illustrating the biological processes associated with each module. **(C)** Enrichment map depicting transcriptional program associations within castration-resistant prostate cancer (CRPC). **(D)** Expression of marker genes for neuroendocrine prostate cancer NEPC in Modules 1-5.

We examined the expression patterns of representative genes within the five transcriptional programs and their associated biological functions and pathways (**Figure 3A**). As the results showed, Modules 1 and 2 were enriched in pathways associated with stress responses to metal ions, detoxification of inorganic compounds, mineral absorption, and copper homeostasis. Module 3 demonstrated a strong correlation with DNA-templated DNA replication, chromosome segregation, and cell cycle pathways. Module 4 exhibited significant enrichment in the epidermal growth factor receptor (EGFR) signaling pathway, the ERBB pathway, endocytosis, and cell-substrate adhesion. Module 5 revealed activation in response to temperature, protein folding, and pathways related to unfolded protein responses. The GSEA enrichment analysis further corroborated these findings.

In the GO enrichment analysis (**Figure 3B**), modules 1 and 2 demonstrated enrichment in pathways central to oxidative stress responses, transmembrane metal ion transport, regulation of systemic homeostasis, and cellular adaptations to arsenic-containing compounds. Module 3 revealed prominent associations with DNA repair and chromatin organization, underscoring its role in safeguarding genomic integrity under conditions of cellular stress. Module 4 was enriched in pathways such as integrin-mediated signaling and actin cytoskeleton regulation, highlighting mechanisms that enhance cellular adhesion, migration, and structural dynamics. Module 5 emphasized the importance of heat shock protein interactions and proteasomal regulation, essential for mitigating proteotoxic stress and maintaining protein homeostasis. Collectively, these pathways illuminate the intricate biological processes underpinning tumor adaptability and progression, further emphasizing the multifaceted roles of these modules in the context of cancer resilience and evolution.

Modules 3, 4, and 5 play critical roles in the development of CRPC (**Figure 3C**). Module 3 drives DNA replication and cell cycle progression, contributing to the rapid proliferation and genomic instability characteristic of CRPC. Module 4 supports tumor survival and migration via EGFR signaling and cell adhesion pathways, enabling tumor adaptation in low-androgen environments. Module 5 enhances the tumor’s ability to withstand therapeutic stress by activating protein folding and stress response mechanisms, allowing CRPC cells to resist treatment. Together, these modules are central to CRPC progression, promoting tumor growth, survival, and adaptation under therapeutic pressure.

In addition, we enrolled NEPC marker from prior studies (25), and compared their expression among the five modules. As showed in **Figure 3D**, module 3 exhibited the higher and more NEPC marker expression, such as NF1A, CPE, COTL1, ETS2, indicating module3 represented a NEPC expression transcriptome program.

### Pseudotime analysis reveals the differentiation patterns of five malignant cell types

**Figure 4:**
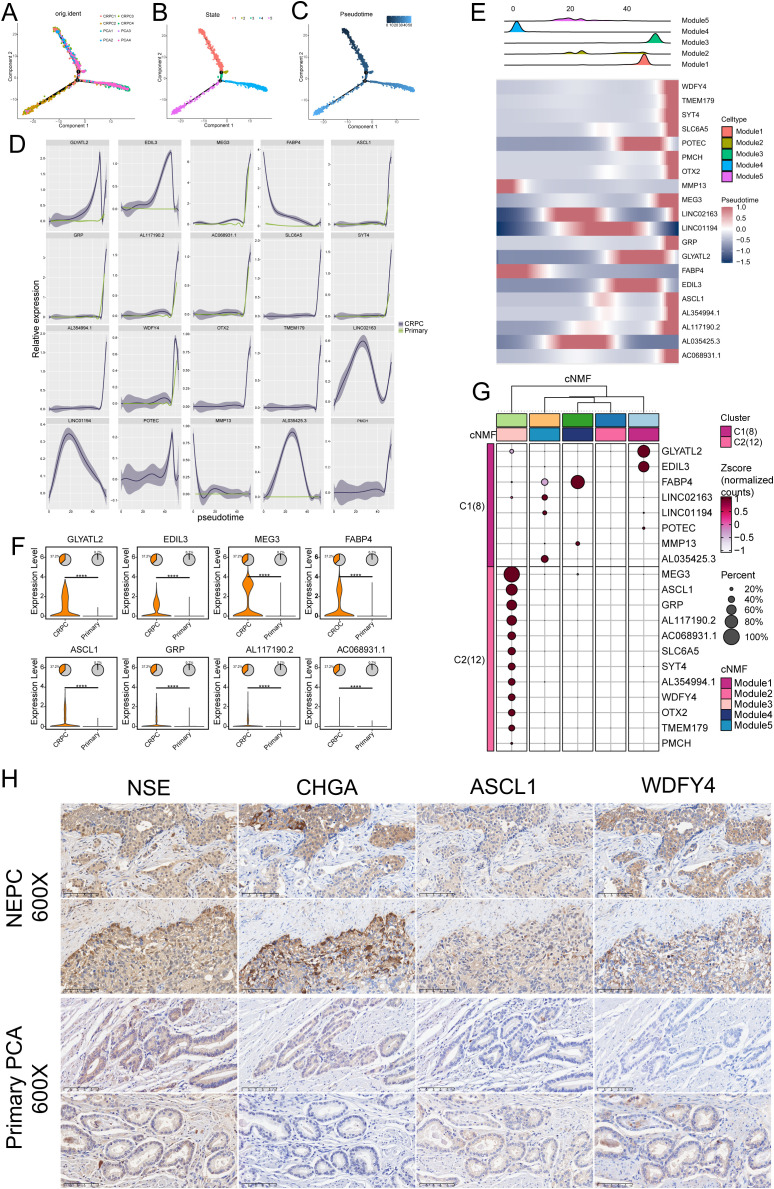
Pseudotime analysis of differentiation patterns in five malignant cell types. **(A–C)** Pseudotemporal analysis using Monocle 2 to explore cell trajectories of prostate cancer (PCa) cells. **(D)** Top 20 trajectory-dependent genes identified in castration-resistant prostate cancer (CRPC) and primary PCa. **(E)** Top 20 trajectory-dependent genes across the five modules. **(F)** Significant differential expression of marker genes between primary PCa and CRPC (****P ≤ 0.0001). **(G)** Clustering analysis showing differential expression of the top 20 marker genes across the five modules. **(H)** Immunohistochemical (IHC) analysis of neuroendocrine prostate cancer (NEPC) and PCa tissues highlighting the differential expression of NSE, CHGA, ASCL1, and WDFY4 between NEPC and primary PCa.

Trajectory analysis revealed a pattern of differentiation trajectories that cluster distinct transcriptional patterns in prostate cancer, modeling the progression from primary PCa to CRPC and NEPC (**Figures 4A-C**). The five identified cell types are categorized into two major fates, with module 3 representing a unique differentiation endpoint. This trajectory is enriched in NEPC cells and is devoid of primary PCA cells, suggesting that these cells may represent the mature NEPC state. Dynamic changes in gene expression along pseudo-time trajectories underscored the top 20 trajectory-dependent genes (**Figures 4D, E**), such as GXYLT2, EDIL3, MEG3, ASCL1, GRP, and WDFY4. These genes exhibit a gradual increase as malignant cells transition from a primary state to a phenotype characteristic of NEPC. These expression trends reflect the molecular alterations accompanying the transition from primary disease to castration-resistant disease and may serve as potential biomarkers of NEPC, warranting further investigation.

Significant differential expression of key genes between primary PCA and CRPC was observed (**Figure 4F**). Compared to primary cancer samples, genes such as GLYATL2, EDIL3, MEG3, FABP4, ASCL1, GRP, and WDFY4 were highly upregulated in CRPC, with statistically significant differences underscoring their potential roles in driving the castration-resistant phenotype.

Additionally, clustering analysis (**Figure 4G**) identified two major clusters, C1 and C2, each representing distinct transcriptional programs. Cluster C1, primarily associated with CRPC, exhibited high expression of genes such as GLYATL2, EDIL3, MEG3, FABP4, and ASCL1. Cluster C2, associated with primary prostate cancer, expressed genes like MMP13 and POTEC. This clustering underscores the distinct molecular programs defining primary and castration-resistant prostate cancer, suggesting that genes highly expressed in Module 3 could serve as specific markers for CRPC.

IHC analysis of prostate tissues was performed to validate the expression of ASCL1 and WDFY4, alongside the established NEPC markers NSE and CHGA. We analyzed tissue samples from two cases of NEPC and two cases of primary PCa. In NEPC tissues, all four markers (ASCL1, WDFY4, NSE, and CHGA) demonstrated strong expression, whereas their expression was markedly reduced in primary PCa samples (**Figure 4H**). These results indicate that ASCL1 and WDFY4 are specifically upregulated in NEPC compared to primary PCa.

### Module 3 type PCa is associated with poorer prognosis

**Figure 5:**
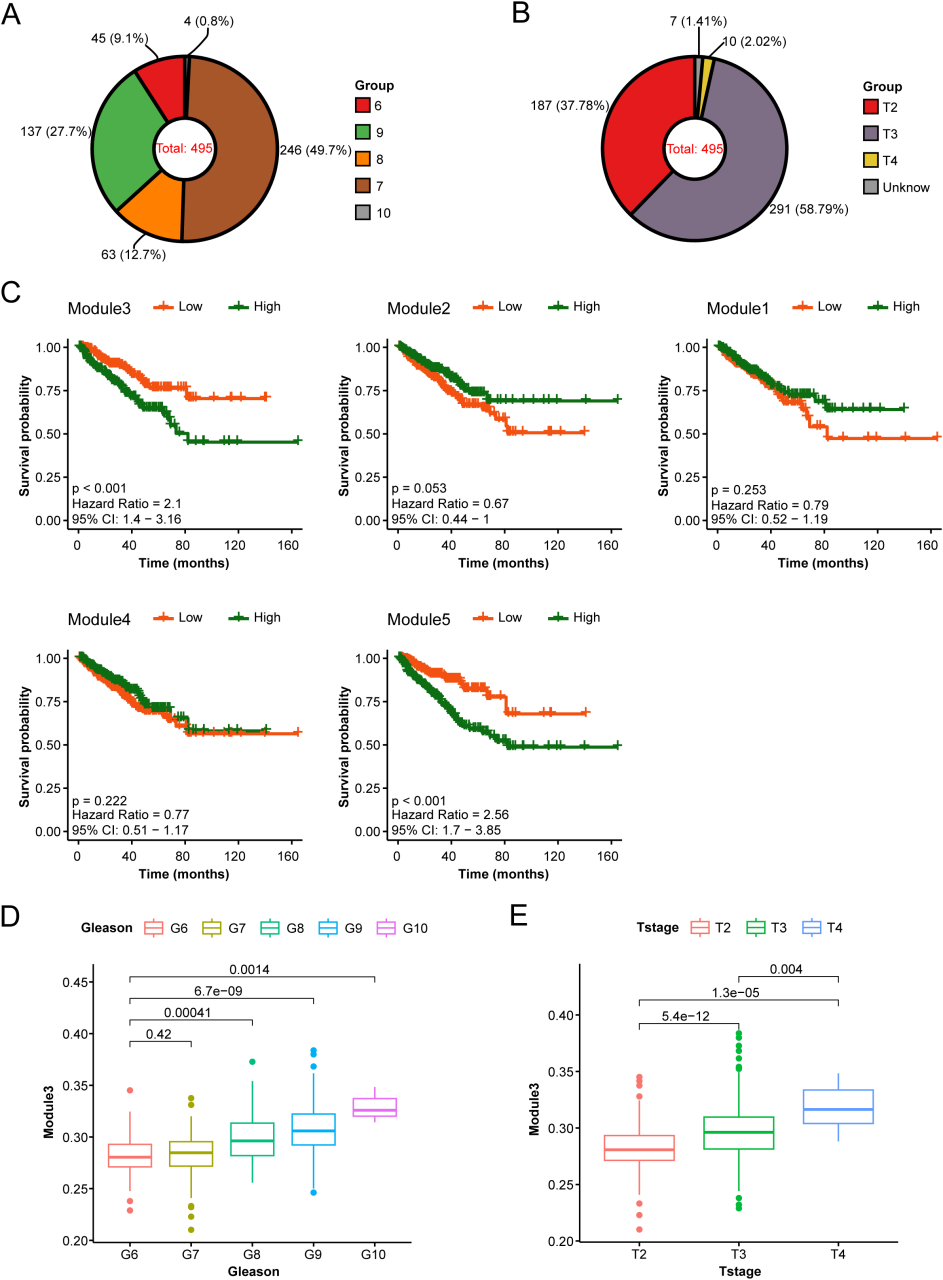
Association of Module 3 with poorer prognosis. **(A, B)** Distribution of Gleason scores **(A)** and T stages **(B)** within the TCGA-PRAD cohort. **(C)** Prognostic analysis of the five modules in the TCGA-PRAD cohort, highlighting the impact of Modules 3 and 5 on patient outcomes. **(D, E)** Proportion of Module 3 across different Gleason scores **(D)** and T stages **(E)**.

Using BayesPrism deconvolution, we estimated the relative contributions of various cell types in prostate cancer samples via Bayesian deconvolution, providing high-resolution insights into cellular composition and distinguishing tumor cells with distinct transcriptional programs. **Figures 5A, B** illustrate the distribution of Gleason scores and T stages within the TCGA-PRAD cohort. Survival analysis indicated that a higher proportion of cells in Module 3 (p < 0.001, HR = 2.1, 95% CI: 1.4~3.16) and Module 5 (p < 0.001, HR = 2.56, 95% CI: 1.7~3.85) was significantly associated with poorer prognosis (**Figure 5C**). Patients with elevated levels of Module 3 had a recurrence risk twice that of patients with low Module 3 levels, while those with high Module 5 levels had a 2.56-fold increased recurrence risk compared to patients with low Module 5 levels.

Additionally, we assessed the proportion of Module 3 across different clinicopathological stages (**Figures 5D, E**). The results showed that Module 3 proportionally increased with rising Gleason scores (all p < 0.05), and a similar trend was observed across increasing T stages (all p < 0.05), corroborating previous findings from earlier analyses.

## Discussion

Our comprehensive analysis of prostate cancer samples provides key insights into cellular heterogeneity and the malignant transcriptional programs driving CRPC progression. By applying advanced single-cell techniques, including consensus non-negative matrix factorization (cNMF) and InferCNV, we dissected the tumor microenvironment and identified transcriptional programs associated with distinct prostate cancer stages, notably CRPC.

Our study identifies key malignant transcriptional programs, particularly Module 3, as crucial drivers of CRPC progression. Module 3, defined by the activation of DNA replication and cell cycle pathways, is specific to CRPC and facilitates rapid cell proliferation and genomic instability, hallmarks of aggressive prostate cancer (26, 27). This aligns with findings linking genomic instability to CRPC via oncogene amplification and loss of tumor suppressors (28). Targeting these proliferative pathways may provide new therapeutic avenues to slow tumor growth and combat treatment resistance.

The pseudotime analysis delineated a trajectory of malignant differentiation, revealing the bifurcation into CRPC and NEPC fates. Notably, Module 3 cells occupied a terminal NEPC state devoid of primary PCa markers, highlighting a unique differentiation endpoint. These results not only elucidate the temporal dynamics of prostate cancer progression but also identify trajectory-dependent genes, such as ASCL1 and WDFY4, as potential biomarkers for NEPC. The gradual increase of these genes along the trajectory underscores their critical role in malignant transformation and their potential as therapeutic targets.

The association between Module 3 and poor prognosis emphasizes its potential as a prognostic marker for CRPC. Higher recurrence risk among patients with elevated Module 3 levels, especially at advanced Gleason scores and T stages, highlights its clinical importance. Genes such as ASCL1, WDFY4, GLYATL2, and EDIL3, which are upregulated in CRPC, likely drive the progression from primary prostate cancer to CRPC (29).

ASCL1 is a pivotal regulator of neuroendocrine differentiation and is crucial in driving the transition from prostate adenocarcinoma to aggressive NEPC, a treatment-resistant subtype of CRPC. ASCL1 is widely recognized as a marker of NEPC. Rodarte et al. demonstrated that ASCL1, while dispensable for the initial formation and growth of PCa, plays an indispensable role in its progression to NEPC. Deletion of ASCL1 effectively abrogates the NEPC transition, instead rerouting the cellular trajectory toward a basal-like phenotype (30). This transcription factor supports lineage-specific changes, allowing prostate cancer cells to adopt neuroendocrine characteristics under therapeutic pressures (31). Studies have found that ASCL1 plays a crucial role in cell proliferation, particularly in neural progenitor cells and oligodendrocyte precursor cells. Both overexpression and loss of ASCL1 can significantly affect cell proliferation behavior (32, 33). Choo et al. suggested that ASCL1 promotes tumor cell proliferation and survival in NEPC by regulating cell cycle-related genes (such as E2F target genes) and neuroendocrine signaling pathways. NEO2734 and BET inhibitors suppress neuroendocrine tumor growth by downregulating ASCL1 expression, thereby inhibiting cell cycle progression (34). This indicates that ASCL1 is not only a key regulator of neuroendocrine differentiation but also plays a crucial role in cell cycle regulation.

In contrast, WDFY4 is a less-characterized gene involved in immune regulation and autophagy (35), and the link between this gene and NEPC remains to be further investigated. CHGA and NSE are well-established neuroendocrine markers extensively studied in the context of NEPC. Their elevated expression levels are commonly associated with NEPC, distinguishing it from typical prostate adenocarcinoma (9). Our findings suggest that ASCL1 and WDFY4 may drive CRPC progression through distinct mechanisms. Given ASCL1’s role in neuroendocrine differentiation, this may indicate a transition to NEPC (30).

Paulo et al. observed the expression of GLYATL2 in PCa cell lines and identified its regulation by the ETV1 transcription factor (36). Similarly, Gasca et al. demonstrated a significant association between high EDIL3 expression and advanced-grade PCa tumors (Gleason score 8–10) using human tissue samples (37). Furthermore, EDIL3 was found to be upregulated in paclitaxel-resistant PC3 cells, whereas its expression was lower in paclitaxel-sensitive LNCaP cells, suggesting a role in chemoresistance. Collectively, these findings validate the upregulation of GLYATL2 and EDIL3 in both human PCa tissues and cellular models.

## Conclusion

Our analysis identified distinct cellular subpopulations within prostate cancer, along with five key transcriptional programs, notably highlighting Module 3 as closely associated with CRPC. Pseudotime analysis traced gene expression dynamics underlying the progression from primary prostate cancer to CRPC. High expression levels of module 3 were significantly associated with poorer prognosis, and the newly identified biomarkers ASCL1 and WDFY4 were validated in IHC analysis. Given that the involvement of ASCL1 in neuroendocrine differentiation and tumor progression suggests that it may be a key driver for the emergence of NEPC. Furthermore, targeting ASCL1 therapy provides an avenue for future exploration of therapeutic strategies for NEPC.

## Data Availability

The datasets presented in this study can be found in online repositories. The names of the repository/repositories and accession number(s) can be found in the article/supplementary material.
