# Supplementary material for: New insights into markers for distinguishing neuroendocrine prostate cancer: evidence from single-cell analysis
Source: Front Immunol. 2025 Mar 14;16:1551815. doi: 10.3389/fimmu.2025.1551815 (PMC11955813; doi:10.3389/fimmu.2025.1551815)
Supplement: Supplementary file 1 [file Image1.pdf]

SUPPLEMENTARY FIGURE 1:

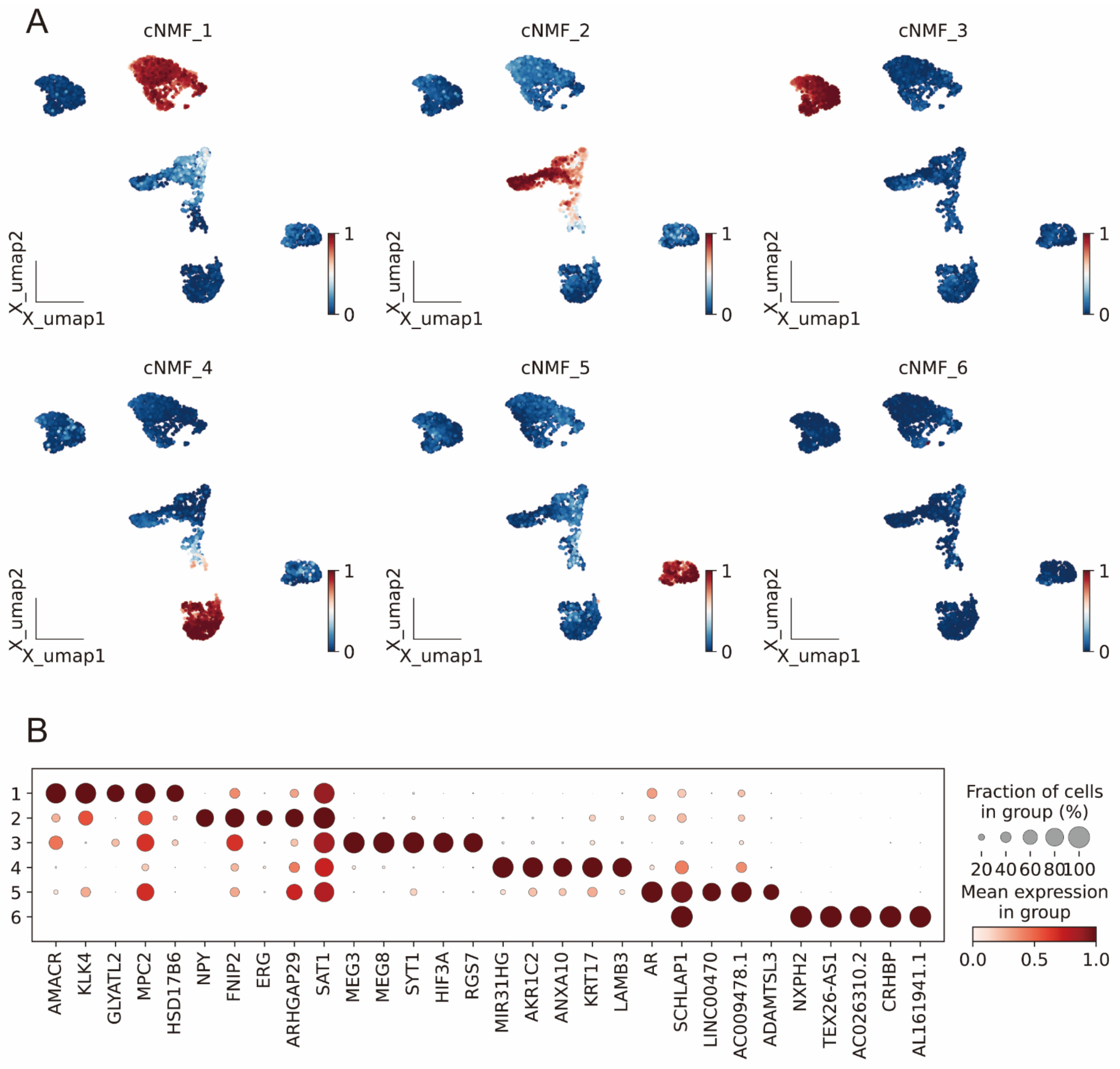

Supplementary Figure 1. (A) UMAP visualization of six malignant transcriptional programs, of which there were too few cells in Module 6 to exclude them from further analysis, resulted in the identification of five key transcriptional programs. **(B)** Dot plot of differentially expressed genes in the 6 modules. Data represent the mean normalized $\log_2(X+1)$ expression scaled from 0 to 1. Dot size represents percent of cells expressing a given gene.

SUPPLEMENTARY FIGURE 2:

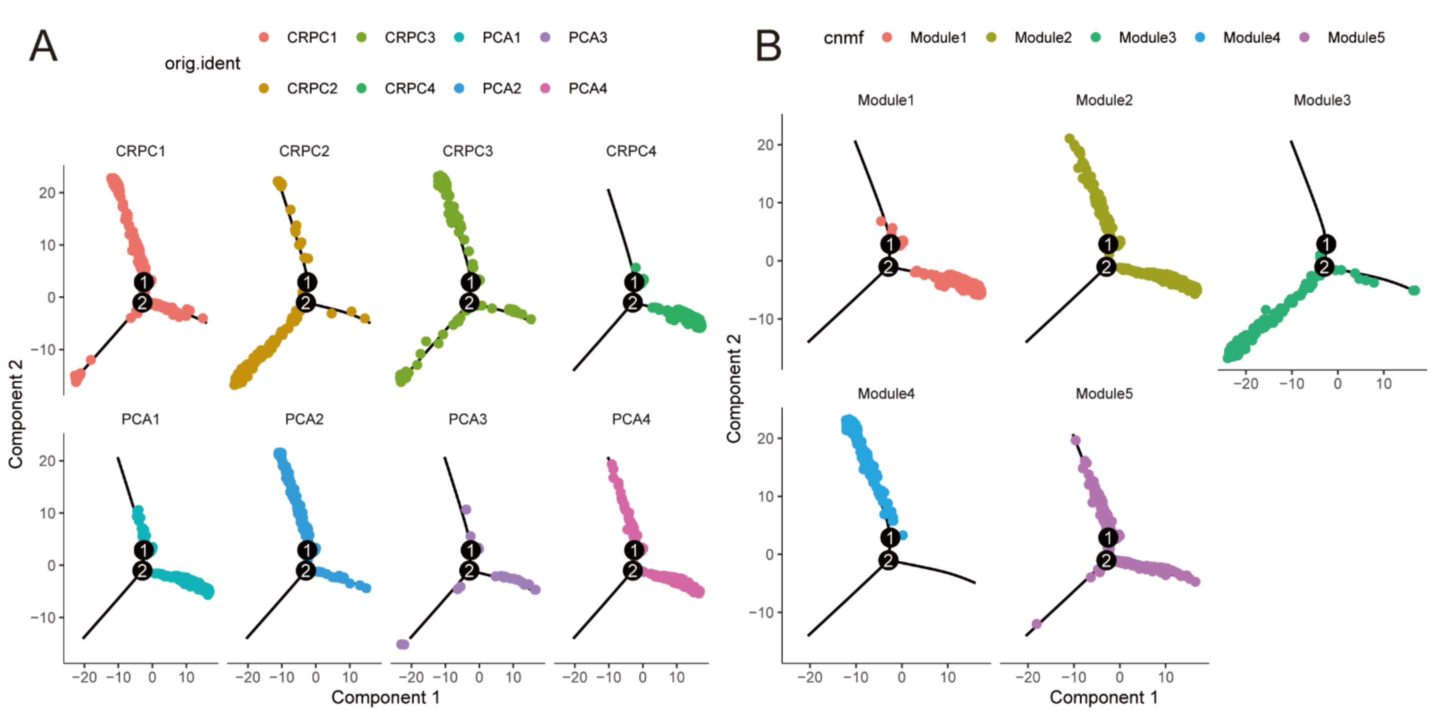

Supplementary Figure 2. Pseudotemporal analysis using Monocle 2 to explore cell trajectories of 8 tumor samples **(A)** and 5 modules **(B)**.

SUPPLEMENTARY FIGURE 3:

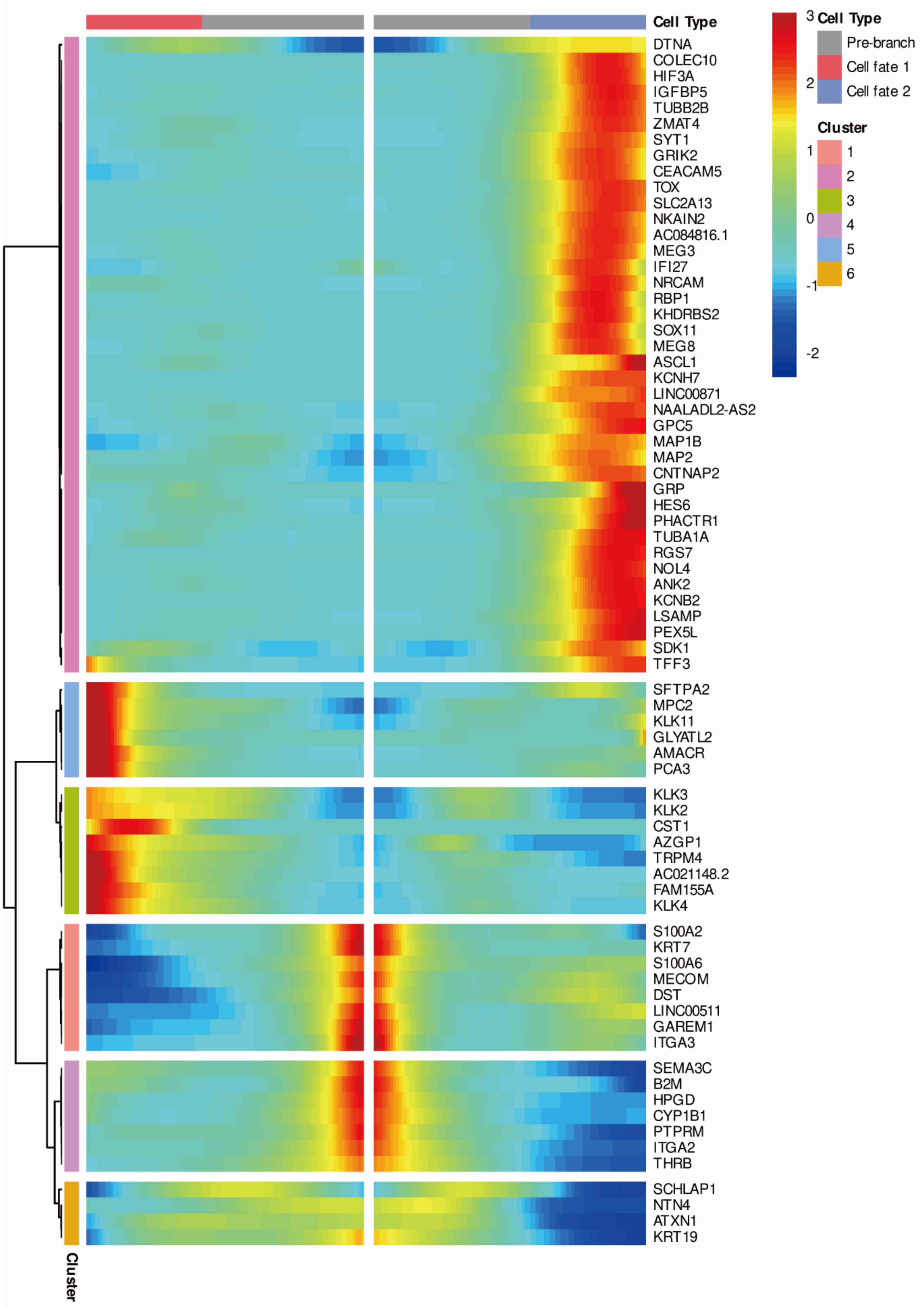

Supplementary Figure 3. Heatmap showing gene expression profiles throughout the pseudotemporal trajectory. Cell clusters transition from a pre-branching state (gray) to two distinct cell fates (cell fate 1 and cell fate 2; red and blue). Genes are clustered according to modules, each of which exhibits a unique temporal expression pattern.
